# Effects of non-surgical decompression therapy in addition to routine physical therapy on pain, range of motion, endurance, functional disability and quality of life versus routine physical therapy alone in patients with lumbar radiculopathy; a randomized controlled trial

**DOI:** 10.1186/s12891-022-05196-x

**Published:** 2022-03-16

**Authors:** Fareeha Amjad, Mohammad A. Mohseni-Bandpei, Syed Amir Gilani, Ashfaq Ahmad, Asif Hanif

**Affiliations:** 1 University Institute of Physical Therapy, Faculty of Allied Health Sciences, The University of Lahore, Lahore, Pakistan; 2 Pediatric Neurorehabilitation Research Center, the University of Social Welfare and Rehabilitation Sciences, Tehran, Iran; 3 University of Lahore, Lahore, Pakistan; 4 Department University Institute of Physical Therapy, Faculty of Allied Health Sciences, Lahore, Pakistan

**Keywords:** Non-surgical spinal decompression therapy, Low back pain, Endurance, Range of motion, Physical therapy, And radiculopathy

## Abstract

**Background:**

Lumbar radiculopathy is an extensively common complaint reported by patients of low back pain (LBP), resulting in several impairments. A comparatively novel technique, non-surgical spinal decompression (NSD), is introduced, which uses a sensitive computerized feedback mechanism and decompresses the spinal nerve roots through segmental distraction. The objective of this study was to determine the effects of NSD therapy in addition to routine physical therapy on pain, lumbar range of motion (ROM), functional disability, back muscle endurance (BME), and quality of life (QOL) in patients with lumbar radiculopathy.

**Methods:**

A total of sixty patients with lumbar radiculopathy were randomly allocated into two groups, an experimental (*n* = 30) and a control (*n* = 30) group, through a computer-generated random number table. Baseline values were recorded before providing any treatment by using a visual analogue scale (VAS), Urdu version of Oswestry disability index (ODI-U), modified-modified Schober’s test (MMST), prone isometric chest raise test, and Short Form 36-Item Survey (SF-36) for measuring the pain at rest, functional disability, lumbar ROM, BME, and QOL, respectively. All patients received twelve treatment sessions over 4 weeks, and then all outcome measures were again recorded.

**Results:**

By using the ANCOVA test, a statistically significant (*p* < 0.05) between-group improvement was observed in VAS, ODI-U, BME, lumbar ROM, role physical (RP), and bodily pain (BP) domains of SF-36, which was in favour of NSD therapy group. The between-group difference was 1.07 ± 0.32 cm (*p* < .001) for VAS, 5.65 ± 1.48 points (*p* < .001) for ODI-U, 13.93 ± 5.85 s (*p* = 0.002) for BME, 2.62 ± 0.27 cm (*p* < .001) for lumbar flexion, 0.96 ± 0.28 (*p* < .001) for lumbar extension, 5.77 **±** 2.39 (*p* = 0.019) for RP and 6.33 **±** 2.52 (*p* = 0.016) for BP domain of SF-36. For these outcomes, a medium to large effect size (d = 0.61–2.47, 95% CI: 0.09–3.14) was observed.

**Conclusion:**

It was concluded that a combination of non-surgical spinal decompression therapy with routine physical therapy is more effective, statistically and clinically, than routine physical therapy alone in terms of improving pain, lumbar range of motion, back muscle endurance, functional disability, and physical role domain of quality of life, in patients with lumbar radiculopathy, following 4 weeks of treatment.

**Trial registration:**

WHO Iranian registry of clinical trials (IRCT20190717044238N1) Dated: 23.12.2019.

## Background

Low back pain (LBP) is one of the most common musculoskeletal disorders [1]. Almost 60 to 80% of adults experience LBP at least once during their life span [2]. Nearly 80% of the structures responsible for LBP pain are associated with intervertebral discs [3], which may accompany radicular symptoms [1, 4]. This radiating pain is called lumbar radiculopathy, which is commonly caused by the compression of lumbar spinal nerve roots and is characterized by burning, electric, or sharp back and leg pain even below knee level into the foot and toes [5, 6]. The prevalence of lumbar radiculopathy is from 3 to 5% in the general population, affecting males more than females (2:1) [6, 7]. Spinal disc herniation is commonly seen in the lumbar region during 25–55 years of age and occurs specifically at L4-L5 and L5-S1 levels [6, 8]. Nerve root compression is typically caused by degenerative intervertebral disc herniation and less commonly by vascular disease, inflammation, congenital abnormalities, infection, or neoplasm [9–11]. For the management of lumbar radiculopathy, several protocols have been used [10].

Most patients respond to conservative treatment, including a few days’ rest, medications, massage, bracing, acupuncture, physical therapy (electrotherapy, heat, traction, spinal manipulation, exercises, etc.) [12]. Still, for a few patients, surgery is recommended [1]. One of the conservative treatment methods for managing lumbar radiculopathy is traction. However, few studies showed that traction therapy might reduce intra-disc pressure and disc protrusion [13]. It may also increase the intervertebral disc space [14] and improve leg mobility [15]. But, a large body of literature has found traction therapy to be an ineffective treatment. Systematic reviews and practice guidelines have declared that probably traction therapy is not superior to sham, placebo, or other treatments, for improving LBP [16–18]. Some problems are associated with ordinal traction, for example, the inability of the patient to tolerate force or position, fatigue, exacerbation of pain, and muscle spasm [19]. However, with recent advances, new technology has been developed, i.e. Non-surgical spinal decompression (NSD) therapy is used to overcome the drawbacks of traction and decompressing the spinal nerve roots non-surgically by using a sensitive computerized feedback mechanism [9, 20, 21]. This system is designed to provide a motorized segmental distraction for a specified time [22] which may induce physical changes to the disc [23, 24]. As a computerized logarithm controls the force of decompression, the NSD device can estimate the actual load that should be applied to the spine and permits the provision of spinal traction force based on this. Consequentially, the muscle tone does not increase excessively [23, 24]. The force is generated within a limit that does not cause muscular or ligamentous stress [25, 26], thus preventing any possible para-spinal muscle spasm [1, 22]. Unlike traction therapy, the NSD technique does not require setting the direction and traction force [24]. It was hypothesized that this technique may also widen the intervertebral space and minimize pressure on the discs by creating a negative pressure in the affected region [21]. It may also reposition the prolapsed disc and restore disc height which is thought to relieve chronic low back pain and other symptoms related to lumbar radiculopathy [21, 27, 28].

Although some of the preliminary data from the literature supported NSD therapy and declared it more effective than conservative treatment methods [9, 22, 26, 29], a few studies have found no superiority of NSD therapy over conservative treatments [21, 30]. Meanwhile, some of the RCTs have confirmed the efficacy of NSD over ordinal traction [19, 24, 31] and declared that NSD is more effective than simple traction in terms of improving back pain, straight leg raise (SLR) [31], radicular pain, disc heights [19], and disc herniation index [24, 31]. On the other hand, there is a lack of high-quality, comprehensive reviews to support the routine use of decompression therapy in clinical practice. To the author’s knowledge, no systematic literature review exists (at the level of Cochrane review) on the efficacy of NSD therapy. Moreover, many studies on spinal decompression therapy have methodological limitations, such as small sample size, lack of blinding, and poor study designs. The results of different studies have also been found conflicting. Furthermore, variable dosages and different patient positions were used in different studies [32]. The present study was an effort towards finding an appropriate spinal decompression technique for lumbar radiculopathy patients. The objective of this study was to determine the effects of non-surgical spinal decompression therapy combined with conventional physical therapy on pain, lumbar range of motion, muscle endurance, and functional disability in patients with lumbar radiculopathy.

## Methodology

### Subjects

This study was a single-blinded, randomized controlled trial conducted over 18 months from 1st January 2020 to 1st June 2021. In this study, sixty patients (28 males and 32 females) aged between 25 and 55 years were selected who were experiencing lumbar radiculopathy. The disease was pre-diagnosed by a neuro-surgeon through clinical examination and X-ray/MRI findings showing disc bulge or prolapsed disc and unilateral radiating LBP for at least 3 weeks. These patients were recruited from an outpatient physical therapy department of Pain Center*,* Lahore, Pakistan. The sample did not include the patients who were reported with a recent fracture or dislocation of the lumbar vertebra, a history of surgery on the lumbar spine, hip or pelvis, spinal infections or tumor in the intervertebral disc, a spinal deformity, any inflammatory disease, spondylolisthesis, osteoporosis below L1, severe disc degeneration or having three or more herniation and pregnant females. An information sheet (explaining the potential risk and benefits of treatment) was provided to the patients. Patients fulfilling the selection criteria and giving written informed consent were recruited in the study, duly approved by the Institutional Review Board (IRB) of the University of Lahore, Pakistan. The demographic details such as height, weight, body mass index (BMI) were also recorded.

### Assessment

The assessment was performed at baseline and after 12 treatment sessions for 4 weeks. Both assessments were performed by an assessor, a qualified and trained physical therapist with more than 5 years’ experience dealing with musculoskeletal disorder patients. The patients were assessed by evaluating their pain intensity at rest, lumbar ROM, trunk extensor muscle endurance, level of disability, and quality of life.

#### Outcome measures

The primary outcomes of this study were pain intensity and lumbar range of motion, while the secondary outcomes were the level of disability, back muscle endurance, and quality of life.

##### Visual analogue scale

The intensity of LBP was evaluated using a visual analogue scale (VAS). The patients were asked to define their present pain intensity at rest by marking a small perpendicular line on a 10 cm horizontal line (‘0’ no pain, and ‘10’ the worst possible pain) [33, 34]. VAS is the most frequently used tool to evaluate pain intensity in patients with LBP. It has shown high validity [33] and reliability [35]. For VAS, the minimum clinically important change and minimum clinically important difference (MCID) were shown 1.1 cm [36] and 1.4 cm [37], respectively, on a 10 cm scale. The minimal detectable change (MDC) for the VAS score was 2 cm (20 mm) [38]. Pain was considered as a primary outcome in this study.

##### Modified-modified Schober’s test

An ordinal tape was used to measure the active lumbar range of motion (ROM) [39]. The examiner marked posterior superior iliac spine PSIS (inferior margins) bilaterally on the fully exposed skin of the patient and drew a horizontal line. A second line was drawn 15 cm above that, and then active lumbar flexion was performed in a pain-free range. The difference between neutral standing and trunk flexion measurements indicates the amount of lumbar flexion. The same method was used for measuring lumbar extension, where patients were instructed to bend backward. Skin marks were wiped out after examination [40]. Testing of lumbar ROM through MMST has demonstrated excellent reliability [39, 41, 42]. Moderate validity with minimum detectable change (MDC) of only 1 cm [42] and minimum clinically important difference (MCID) of greater than 1 cm was noted [43]. The lumbar ROM was considered as a primary outcome.

##### Oswestry disability index

ODI is a ‘gold standard’ self-administered questionnaire for assessing low back functional disability [44]. It contains ten sections involving pain intensity, social and sex life, and different personal activities. Each section has six possible answers to be marked on a 0 to 5 scale. The higher scores indicate a higher level of functional disability. The total score is 50, usually represented as a percentage [45]. A single agreed-upon minimal clinically important difference (MCID) score has not been recognized yet for the ODI. The MCID of ODI varies, such as 17 points, 10 points, 6 and 5 point change [46]. The Urdu version of the Oswestry disability index (ODI-U) was used, with good to moderate validity and excellent reliability in patients with lumbar radiculopathy. The MDC of approximately 6 points has been reported on a 0–50 scale for lumbar radiculopathy patients [47]. The level of disability was considered as a secondary outcome.

##### Prone isometric chest raise test

The isometric endurance of back extensor muscles was assessed through a “prone isometric chest raise test,” as described by Ito et al. [48]. The patient was lying prone with arms along the sides. A small pillow was placed under the abdomen to decrease the lumbar lordosis [49]. The patient was requested to lift the sternum about 30 degrees off the couch to maintain maximum cervical spine flexion and gluteal muscle contraction for pelvic stabilization. The patients were instructed to maintain the position as long as possible but not exceed the five-minute time limit. The examiner recorded the time duration in seconds using a stopwatch while the chest was kept off the couch [48, 50, 51]. It is a highly reliable [48] and valid test to evaluate the endurance of trunk extensor muscles with an MDC of around 19 s [48, 51]. No information was found in the literature regarding MCID of prone isometric chest raise test. The back muscle endurance was considered as a secondary outcome.

##### RAND short form 36-item health survey (Sf-36)

RAND Short Form 36-item questionnaire (SF-36) was used to assess the patients’ quality of life. It has eight subscales, including Physical Functioning (PF), Role Limitation Due to Physical Health (RP), Role Limitation Due to Emotional Problems (RE), Energy and Fatigue/Vitality (VT), Emotional Well-being/Mental Health (MH), Social Functioning (SF), Body Pain (BP), and General Health (GH). The score in each subscale is converted into a 0–100 scale ranging from worst to best [52]. RAND SF-36 scoring method provides eight domain score with no physical component summary (PCS) and mental component summary (MCS) [53]. However, SF-36 total scoring is discouraged by both the developers and the SF-36 scoring manual [54]. SF-36 was found to be a valid [55], responsive [56], highly reliable, and internally consistent scale for assessing the health status and quality of life in LBP patients with MDC of only 20 points [57] on its all eight domains [58–60]. The quality of life was considered a secondary outcome.

##### Randomization

**Fig. 1:**
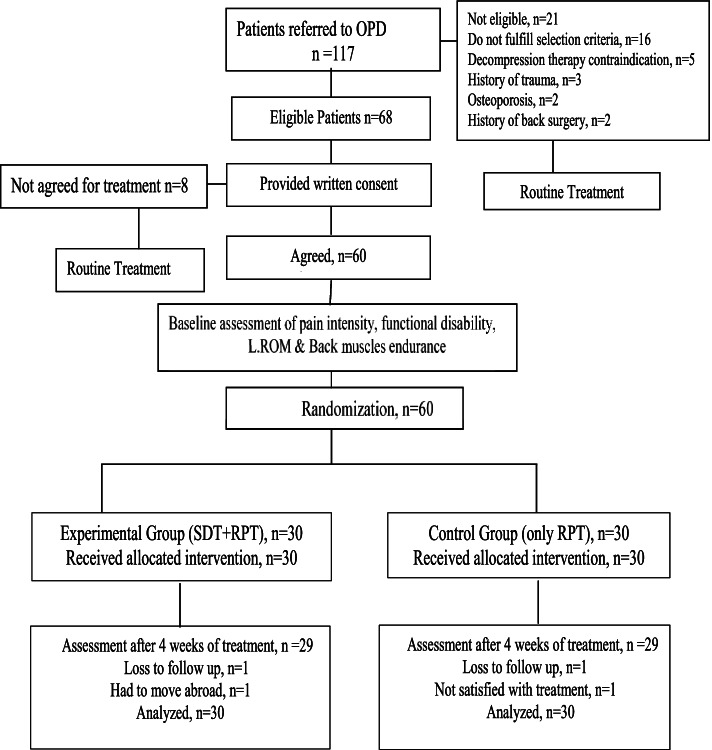
Flow diagram showing recruitment and assessment of patients. NSD = Spinal Decompression Therapy, RPT = Routine Physical Therapy, L.ROM = Lumbar Range of motion

Randomization was carried out using a computer-generated random number table through a simple random sampling technique. Those numbers were sealed in envelopes opened by the main investigator to assign the allocated treatment. Sixty eligible patients were allocated to an experimental (spinal decompression therapy) group and a control group (routine physical therapy). The process of participants’ assignment to these groups is represented in the CONSORT flow diagram (Fig. 1).

##### Blinding

This study was a single-blinded, randomized controlled trial. The outcome assessor (specialized in musculoskeletal physical therapy and had more than 5 years of experience dealing with back pain) was blinded to the allocation of patients to the groups.

### Interventions

Patients in the experimental and the control groups received routine physical therapy treatment. However, spinal decompression therapy was applied to patients in the experimental group in addition to this. Both groups received the treatment for 4 weeks. Each patient completed a total of twelve treatment sessions (i.e., three sessions per week on alternate days at the physical therapy outpatient department). Sixty patients (30 in each group) were assigned randomly to the following groups:

- Control group- Routine Physical Therapy Group:

Routine physical therapy treatment included mild stretching of tight muscles and nerves, core stabilization exercises [61], ergonomic and postural awareness, as well as the pain relief modalities [30, 62, 63], i.e., TENS (Pre-Programmed Dual Channel TENS 120Z; ITO CO., LTD. Japan), ultrasound (US Pro 2000, USA) and hot pack. The TENS was applied in a conventional mode for 15 min at a high frequency of 70 Hz and wavelength of 100 microseconds [63] by placing two 40 × 40 mm electrode sets cross arranged on each side of the lumbosacral spine [64]. The ultrasound was applied over the painful lumbosacral area with circular movements of the probe head for 5 min using the intensity of 1.3 W/cm2 and frequency of 1 MHZ in continuous mode [63–65].

b) Experimental group-Spinal Decompression Therapy Group

Along with the treatment provided to the control group, patients in the experimental group also received spinal decompression therapy through the SPINE MT device (Shinhwa Medical, Gimhae, Korea) for 20 min. A computer regulated the whole system of the device. The machine had an air pumping sac (inflated to maintain the lumbar spine lordosis) [24], disc-angle-pull adjusters (provides level-specific decompression) [1, 22], and harnesses (to fix the upper and lower body) [1]. A pelvic tilt angle of 15 and 10 degrees was set for patients with intervertebral disc herniation at L4-L5 and L5-S1 levels, respectively [24]. First, the patient’s demographic information was entered into the computer. Then, the disc level to be treated was selected based on the patient’s clinical condition and MRI findings. The patient attained a supine lying position on a motorized split-table, with a movable lower half. Adjustable thoracic and pelvic belts were placed on the patient’s body. Support was adjusted under the patient’s knees to reduce pelvic rotation. The patient was instructed to push the safety button any time, spontaneously eliminating the whole tension. The decompression force was set according to the patient’s tolerance with an initial pulling force of almost 25% of the patient’s body weight, which was increased per session with a final pulling force of 50%. If a patient felt decompression pull to be painful or strong, the distraction force was reduced up to 10–25% [1, 3]. The ratio of distraction and relaxation (hold and rest) was set at 60:30 s (2:1 ratio) [66].

### Sample size

The sample size was calculated to satisfy outcomes for both pain (VAS) and disability (ODI). For VAS the minimum total sample was 24 (alpha = 0.05, power = 0.8, target difference = 1.9) [1*].* For ODI the minimum total sample was 48 (alpha = 0.05, power = 0.8, target difference = 13.7) [2*].* Therefore, allowing for 25% dropout, 60 patients were included in the study.

### Data analysis

The data were tabulated and analyzed using SPSS (version 21). Descriptive statistics, including mean and standard deviation, were calculated for each variable. Assumptions of normality were checked using the Kolmogorov test, which indicated no apparent violation of the assumption (*P* = 0.054–2.00). After controlling pre-test scores, analysis of covariance (ANCOVA) was used to compare between-group changes and evaluate which group received the more effective intervention. The *P*-value was considered significant when it was less than 0.05, and the confidence interval was kept at 95%. Cohen’s d was used to evaluate the effect size between the two groups. The effect size of 0.2, > 0.5, and > 0.8 were considered small, medium, and large, respectively [67].

## Results

A total of 117 patients were referred to the outpatient physical therapy department over 18 months. Sixty-eight patients were found eligible according to the selection criteria. Eight patients were unwilling to participate in the study, and the remaining 60 patients were randomly and equally divided into two groups. In both groups, two patients were not available for the post-treatment assessment. One of these patients had to move abroad, and one was not satisfied with the treatment. The withdrawal details are mentioned in Fig. 1. However, these missing patients were involved in post-treatment analysis by calculating the group mean for missing data [68]. In Table 1, the baseline characteristics of all participants are summarized.

**Table 1:** Baseline Characteristics of the Patients

Variables	Exp. Group (*n* = 30) Mean ± SD	RPT Group (*n* = 30) Mean ± SD	Total (*n* = 60) Mean ± SD
Age (Years)	39.57 ± 5.16 (29–55)^a^	43.70 ± 10.48 (25–55)^a^	41.63 ± 8.45
Height (cm)	166.37 ± 10.74	167.23 ± 9.68	166.80 ± 10.14
Weight (kg)	74.20 ± 13.64	72.67 ± 8.34	73.43 ± 11.24
BMI	26.95 ± 5.29	26.19 ± 3.97	26.57 ± 4.65
VAS (0–10)	5.55 ± 0.97	5.18 ± 1.29	5.37 ± 1.15
ODI (0–100)	31.28 ± 11.34	32.27 ± 10.33	31.77 ± 10.76
BME (seconds)	18.94 ± 8.76	21.18 ± 5.77	20.06 ± 7.44
ROM (cm) Flex	3.72 ± 0.81	3.31 ± 0.77	3.51 ± 0.81
ROM (cm) Ext.	1.73 ± 0.78	1.58 ± 0.59	1.66 ± 0.69
SF-36-PF	50.43 ± 17.33	49.67 ± 9.91	50.05 ± 14.00
SF-36-RP	51.50 ± 8.84	47.53 ± 9.09	49.52 ± 9.11
SF-36-BP	38.20 ± 9.56	39.08 ± 10.01	38.64 ± 9.71
SF-36-GH	28.50 ± 7.89	30.47 ± 12.60	29.48 ± 10.47
SF-36-VT	51.58 ± 19.25	45.80 ± 12.50	48.69 ± 16.35
SF-36-SF	44.50 ± 16.05	44.33 ± 14.49	44.42 ± 15.16
SF-36-RE	47.50 ± 9.45	49.00 ± 10.31	48.25 ± 9.83
SF-36-MH	55.07 ± 19.95	56.80 ± 13.22	55.93 ± 16.80

*RPT* Routine Physical Therapy, *BMI* Body mass index, *VAS* Visual analogue scale, *ODI* Oswestry disability index, *BME* Back muscle endurance, *ROM* Range of motion, ^a^Values in parentheses are range, *Flex* Flexion, *Ext.* Extension, *SF-36* Short Form 36 item survey, *PF* Physical Functioning, *RP* Role Limitation due to Physical Problems, *BP* Bodily Pain, *GH* General Health, *VT* Vitality, *SF* Social functioning, *RE* Role limitation due to Emotional Problems, *MH* Mental health

### Back pain intensity

In both groups, the intensity of back pain at rest was significantly decreased after treatment. However, there was more improvement in the experimental group than the control group, with a mean change of 3.61 ± 1.27 vs 2.31 ± 0.96 points on VAS. The mean difference of VAS was also significant between groups and was in favour of the experimental group (*P* < .001, F (1,57) =28.503). Moreover, after 4 weeks, a large effect size (d = 1.07, 95% CI: 0.53–1.61) for VAS was shown between two groups that favoured the experimental group (Table 2).

**Table 2:** Between Group Differences and Effect Size for Pain, Functional Disability, BME, Lumbar ROM and Quality of Life

Variables	Exp Group Mean ± SD	RPT Group Mean ± SD	Mean Difference Mean ± SD	Effect Size (95% CI)	***P***-value
VAS (0–10)	2.56 ± 1.05	3.63 ± 0.94	1.07 ± 0.32	1.0**7** (0.53–1.61)	< 0.001*
ODI (0–100)	16.56 ± 4.95	22.20 ± 6.41	5.65 ± 1.48	0.98 (0.45–1.52)	< 0.001*
BME (sec.)	59.46 ± 27.24	45.53 ± 16.88	13.93 ± 5.85	0.61 (0.09–1.13)	0.002*
ROM (Flex)	6.92 ± 0.90	4.30 ± 1.20	2.62 ± 0.27	2.47 (1.79–3.14)	< 0.001*
ROM (Ext.)	3.53 ± 1.35	2.57 ± 0.78	0.96 ± 0.28	0.87 (0.34–1.40)	< 0.001*
SF-36-PF	59.00 **±** 7.81	53.87 **±** 17.44	5.13 **±** 3.49	0.38(−0.13–0.89)	0.154
SF-36-RP	64.27 **±** 9.45	58.50 ± 9.09	5.77 **±** 2.39	0.622 (0.10–1.14)	0.019*
SF-36-BP	52.50 **±** 9.98	46.17 **±** 9.54	6.33 **±** 2.52	0.649 (0.13–1.16)	0.016*
SF-36-GH	31.87 **±** 8.71	32.07 ± 7.18	0.20 ± 2.06	0.025(−0.48–0.53)	0.923
SF-36-VT	53.72 **±** 17.07	48.33 **±** 13.94	5.39 **±** 4.02	0.346(−0.16–0.85)	0.333
SF-36-SF	56.50 **±** 8.44	54.83 **±** 9.96	1.67 **±** 2.38	0.181(−0.32–0.68)	0.469
SF-36-RE	55.17 **±** 7.48	57.17 ± 7.48	1.70 **±** 1.99	0.22(−0.28–0.72)	0.310
SF-36-MH	59.33 **±** 10.97	57.33 **±** 15.87	2.00 **±** 3.52	0.147(−0.36–0.65)	0.573

*RPT* Routine Physical Therapy, *VAS* Visual analogue scale, *ODI* Oswestry disability index, *BME* Back muscle endurance, *ROM* Range of motion, *Flex* Flexion, *Ext.* Extension, *SF-36* Short Form 36 item survey, *PF* Physical Functioning, *RP* Role Limitation due to Physical Problems, *BP* Bodily Pain, *GH* General Health, *VT* Vitality, *SF* Social functioning, *RE* Role limitation due to Emotional Problems, *MH* Mental health

*P*-value less than 0.05 was considered significant (*)

### Back functional disability

The functional disability of the back was significantly reduced from pre to post-intervention in both groups. However, patients in the experimental group showed more improvement than the control group, with a mean change of 14.72 ± 13.11 vs 10.32 ± 10.32 points on ODI-U. Furthermore, a significant difference in ODI-U change scores between groups was found after 4 weeks, which was in favour of the experimental group (*p* < .001, F (1,57) =17.260). A large effect size (d = 0.98, 95% CI: 0.45–1.52) was also seen after 4 weeks for ODI-U between the two groups favouring the experimental group. (Table 2).

### Back muscle endurance

In both groups, back muscle endurance was increased significantly after treatment. However, there was more improvement in the experimental group than the control group, with a mean change of 42.55 ± 28.28 vs 24.32 ± 17.53 s. as measured through a stopwatch. The mean difference of BME was also significant between the groups and favoured the experimental group (*p* = .002, F (1,57) =10.65). Moreover, after 4 weeks of intervention, a medium effect size (d = 0.61, 95% CI: 0.09–1.13) for BME was shown between the groups that favoured the experimental group (Table 2).

### Lumbar ROM

Lumbar ROM was improved significantly in both groups from baseline to post-treatment. The patients in the experimental group presented more improvement than the routine physical therapy group with a mean change of 3.21 ± 0.67 cm vs 0.77 ± 1.40 cm in flexion and 1.80 ± 934 cm vs 0.99 ± 0.81 cm in extension when measured through MMST. The between-group analysis presented that lumbar ROM was improved significantly, including flexion (*p* < .001, F (1,57) =93.43) as well as extension (*P* < .001, F (1,57) =12.23) noted after 4 weeks’ intervention. Furthermore, the large effect size for flexion (d = 2.47, 95% CI: 1.79–3.14) and extension (d = 0.87, 95% CI: 0.34–1.40) was revealed between the groups after the intervention of 4 weeks in favor of the experimental group (Table 2).

### Quality of life

Following 4 weeks of treatment, the RP, BP, SF, and RE domains of quality of life were significantly (*p* < 0.05) improved in both groups. Additionally, the PF domain was also significantly (*p* = 0.005) improved in the experimental group. The patients in the experimental group presented more improvement than the routine physical therapy group with a mean change of 12.77 ± 1.14 vs 10.97 ± 0.41 points in RP, 14.30 ± 13.05 vs 7.08 ± 14.73 points in BP, 12.00 ± 16.03 vs 10.50 ± 13.15 points in SF, and 9.67 ± 9.26 vs 6.47 ± 9.98 points in RE domains of QOL. Bot no statistically significant (*p* > 0.05) improvement was observed in both groups in the GH, VT, and MH domains of QOL. After between-group analysis, a significant mean difference was observed for RP (*p* = 0.019, F (1,57) =60.27) and BP domain (*p* = 0.016, F (1,57) =6.17) which was in favour of the experimental group. However, no statistically significant (*p* > 0.05) between-group improvement was observed in PF, GH, VT, SF, RE, and MH domains of SF-36. Moreover, after 4 weeks, the medium effect size for RP (d = 0.622, 95% CI: 0.104–1.140) and BP (d = 0.649, 95% CI: 0.130–1.168) domains was also seen between groups that favoured the experimental group. (Table 2).

## Discussion

The current research study aimed to evaluate the effects of non-surgical spinal decompression therapy in addition to routine physical therapy versus routine physical therapy alone on pain, range of motion, endurance, functional disability, and quality of life in patients with lumbar radiculopathy. According to between-group analyses, more statistical improvement was observed in the experimental group regarding pain intensity, functional disability, lumbar ROM, BME, RP, and BP domains of QOL compared to the control group. Moreover, a medium to large effect size (d = 0.61–2.47) was observed for VAS, ODI-U, BME, LROM, RP, and BP domains of QOL, favouring the experimental group. The magnitude of effects in the present study is larger and inconsistent with previous systematic reviews of simple traction therapy, which has found traction to be ineffective [16–18]. As explained in those studies, the reason could be mixed groups of patients, varying levels of activity, varying degrees and stages of the disease, and a broad spectrum of parallel therapies [69]. Regarding the efficacy of NSD, no high-quality systematic review exists. However, some of the comparative RCTs conducted on the effectiveness of NSD and simple traction therapy have declared NSD therapy superior to ordinary traction [19**,**^24^**,**^31^**].** in terms of improving back pain, SLR [31], radicular pain, disc heights [19], and disc herniation index [24, 31].

The current study results are consistent with a few previously conducted RCTs that show NSD therapy provides better results when applied with other conservative treatment methods [9, 22, 26, 29]. In an RCT, Oh et al. (2017) found NSD therapy to be more effective for improving pain and functional status of CLBP patients as compared to the conservative treatment received by the control group, which included hot-pack, infrared, and ultrasound, while the experimental group received NSD therapy in addition to the conservative treatment for 4 weeks [26]. In another RCT conducted by Shah et al. (2020), the experimental group showed more improvement in the walking duration of lumbar radiculopathy patients than the control group. The experimental group received NSD therapy, TENS, heating, and exercises (strengthening and stability) for 4 weeks. The same treatment was provided to the control group, except NSD therapy [9]. The treatment duration, nature of treatment received by both experimental and control groups, and the study findings were comparable to the present study. However, the back muscles endurance and lumbar ROM were the additional outcome measures not studied previously. Moreover, Gaowgzeh et al. (2020) conducted a single-blinded RCT to determine the effects of spinal decompression therapy and core stabilization exercises (CSE) on chronic Lumbar disc prolapse (LDP) patients treated for 6 weeks. The results indicated that spinal decompression therapy and CSE are more effective than interferential therapy and CSE in reducing pain and the functional disability of patients with chronic LDP [22]. A comparison of these findings with the present study indicates more time duration and different conventional treatments. However, these findings are still comparable to the present study, indicating that the same effect may be obtained in less time with treatment combinations used in the present study.

However, a few studies found no superiority of NSD therapy over conservative treatment methods [1, 3, 21, 30]. In an RCT, Demirel and colleagues (2017) found that NSD therapy was not superior to conservative treatment (electrotherapy, deep friction massage, and exercise) in improving pain, function, and herniation thickness. However, disc herniation size was improved more in the NSD therapy group over 6 weeks of treatment [30]. Two of the outcomes, i.e., herniation size and thickness (measured through MRI), were out of the scope of the present study. In contrast, the reason for no improvement in other outcomes could be a small sample size (*n* = 20), the large age range of patients (25–65 years), and the sub-acute stage of disease (pain for 8 weeks). In an RCT by Choi et al. (2015), no statistically significant difference between NSD and general traction was found. Both groups received hot-pack, ultrasound, infrared current. NSD and traction had the same effects on improving pain, disability, and straight leg raise in patients with intervertebral disc herniation over 4 weeks [3]. The effect of these two types of equipment on lowering intra-discal pressure in the lumbar region could be responsible for improving the patient’s symptoms. However, in the current study, the pain levels and pain-related functions were also improved in the control group. It might have occurred because the conventional treatment might have reduced the inflammatory mediators and released nerve growth factors. Previous literature has revealed that annular ruptures cause inflammatory mediators to rise and release nerve growth factors which induce pain [70]. In another RCT by El-Gendy et al. (2015), the effect of non-surgical spinal decompression therapy on chronic lumbar disc prolapse (CLDP) patients was assessed. The experimental group received NSD, icing, McKenzie, and stability exercises, while the control group received only exercises and ice. NSD therapy was effective clinically in pain reduction, but no significant difference was observed statistically after 6 weeks of treatment [21]. The reason could be the involvement of a very limited number of patients. Further, the study included only male patients, which reduced the extent of generalization of the results.

The pain reduction in both groups was also meaningful clinically, as it was greater than MCID of the VAS scale, i.e., 1.4 cm [36]. The control group showed less improvement than the experimental group with an MCID of 2.31 cm (95% CI: 1.96–2.66) vs 3.61 cm (95% CI: 3.15–4.07) after 4 weeks of treatment. This finding is consistent with the studies of Lee et al. [66] and El-Gendy et al. [21], who found a clinically significant difference between groups with MCID of 3 cm (95% CI: 2.20–3.74) in the NSD therapy group vs 1.28 cm (95% CI: 0.54–1.86) in the control group after 4 weeks and 4.88 cm (95% CI: 4–6.23) in NSD therapy group vs 2.78 cm (95% CI: 1.34–4.84) in the control group after 6 weeks, respectively on a 10 cm VAS scale. Contrary to the findings of the present study, Choi et al. [3] and Kocak et al. [1] found no clinically significant difference between groups with MCID of 1.9 cm (95% CI: 0.89–2.87) in the NSD therapy group vs 1.1 cm (95% CI: 0.21–2.01) in the control group after 4 weeks and 2.5 cm (95% CI: 1.55–3.43) in NSD therapy group vs 2.3 cm (95% CI: 1.37–3.25) in the control group after 6 weeks, respectively on a 10 cm VAS scale. A potential reason may be the lack of a control group in the study, which is necessary to determine the real effects of treatment.

The present study’s findings revealed that improvement of the functional status of the lumbar spine in the decompression group was greater than MCID for ODI, which is not similar in studies, with the variation of 5, 6, 10, or 17 points on 50 point scale [46]. Kocak et al. [1] and Ma et al. [71] found MCID of 13.70 points (95% CI: 9.26–18.64) and 17.33 points (95% CI: 10.84–24.32) in ODI score after 6 weeks and 4 weeks of decompression therapy, respectively. These results are comparable with the present study’s findings, where the MCID for ODI is 14.72 points (95% CI: 10.29–19.15) after 4 weeks of decompression therapy. On the contrary, Choi et al. [3] found a slightly lower mean change of 9.80 points (95% CI: 0.64–18.74) after 4 weeks, on ODI score in the NSD therapy group. In contrast, El-Gendy et al. [21] and Demirel et al. [30] observed a greater mean change of 40 points (95% CI: − 9.58-69.52) and 42 points (95% CI: 31.71–49.22), respectively after 6 weeks. It is probably due to the long treatment duration of 6 weeks instead of 4 weeks treatment provided in the present study showing that lengthening the treatment period or increasing the number of treatment session may yield diverse findings. As the studies were gender specific with very small sample size (*n* = 20), their results could be uncertain and could not be generalized.

The current study showed more improvement in the experimental group in terms of lumbar flexion and lumbar extension with MCID of 3.21 cm (95% CI: 2.77–3.63) and 1.80 cm (95% CI: 1.24–2.36), respectively, which is greater than the MCID of modified-modified Shober’s test (> 1 cm) [43]. Lee et al. [66] and Mobeen et al. [72] observed an increase in lumbar ROM in the NSD therapy group after four and 2 weeks of treatment using a 3D motion analyzer and universal goniometer, respectively. None of the studies on the effects of NSD therapy assessed lumbar ROM through MMST, which is a highly reliable and valid test and an easy, fast, safe, and inexpensive test to be used clinically [73]. On the other hand, in most studies, straight leg raise (SLR) was assessed instead of directly measuring the lumbar ROM [3, 21, 30, 31, 64, 71]. However, measuring lumbar ROM in patients with lumbar radiculopathy is of great importance [74, 75].

The present study showed more improvement of BME in the experimental group with an MCID of 42.55 s (95% CI: 32.45–52.65) compared to the control group with an MCID of 24.32 s (95% CI: 17.93–30.71). To the authors’ knowledge, no study has observed the effects of NSD therapy on back muscle endurance to date. However, trunk extensor muscle endurance has been identified as a good back health predictor in LBP patients, and back muscle endurance is usually reduced in LBP patients [50, 76, 77]. Therefore, BME was considered as an outcome in the present study.

The current study revealed a statistically significant between-group improvement in the physical role and bodily pain domains of QOL. However, the control group showed less improvement than the experimental group with MCID of 10.97points (95% CI: 10.82–11.11) vs 12.77points (95% CI: 12.36–13.17) in RP and 7.08points (95% CI: 1.81–12.35) vs. 14.30points (95% CI: 9.63–18.97) in BP domain. Although it is believed that decreased pain, increased ranges, functional status, and endurance would lead to a sense of well-being and improved quality of life. But no statistically significant (*p* > 0.05) between-group improvement was observed in PF, GH, VT, SF, RE, and MH domains of QOL. The reason behind this non-achievement could be that post-treatment measurements were taken straightway after the four-week intervention, which was too early to improve the patients’ quality of life. A long-term follow-up may show improvement in these domains too. Kocak et al. (2018) declared that NSD is not superior to conventional traction in reducing pain and depression and improving functionality and quality of life [1]. To the author’s knowledge, only the present study observed the quality of life (using SF-36) of the patients receiving NSD therapy. Contrary to the present study’s findings, Kocak et al. found no statistically significant within-group differences in SF-36 scores except for the RP domain in the NSD group and BP domain in the conventional traction group after 6 weeks of treatment. The reason could be that; the study lacks a combination of therapies which is quite necessary because it is unethical to apply only traction to the patients with chronic pain and is also not according to the manufacturer treatment protocol guidelines, which recommend the application of heating or myofascial release, muscle stimulation and stretching exercises before NSD treatment [1]. While treating the patients with lumbar radiculopathy, the clinicians must consider the lack of high-level evidence, as no Cochrane-level systematic review (to the author’s knowledge) exists on the effectiveness of NSD on such patients.

The current study also has some limitations, which must also be considered, such as the additional therapy time was given to the interventional group compared to the control group. The “high-technology” intervention and additional therapy time vs control may have significantly impacted patient-reported outcome measures (PROMs) and led to the potential Hawthorne effect. Moreover, due to the nature of the treatment, it was not possible to maintain patients’ blinding, which may also have caused the Hawthorne effect. Another limitation was that, although the outcome assessor was blinded regarding the type of intervention given to the patients, the extent to which the assessor remained blinded was not assessed. The patients and the main investigator were instructed not to disclose the allocation status to the assessor at any stage. Due to lack of resources, the prone isometric chest raise test was used, instead of surface EMG, etc. However, this is also a valid and reliable test to assess trunk muscle endurance and may cause no potential effects on the study results. The lack of follow-up after therapy ceased was another limitation. Since, due to the prevailing Covid-19 pandemic, it was difficult to recruit patients post-treatment for a follow-up assessment to perform the objective measurements. Therefore, only the short-term effects of the relevant treatment were assessed. Moreover, some of the therapies provided to the control group were not guideline-based; instead, they are commonly used and accepted control therapies in Pakistan but not internationally.

The present study’s findings revealed that choosing this management strategy may assist health care professionals in improving patient symptoms in less time and decreasing the economic burden of LBP on society by treating the patients non-surgically. Successful non-surgical treatment is less than a tenth of the cost of surgery [78]. Long-term outcome studies with almost one-year follow-up are recommended for further investigation if non-surgical decompression therapy prevents future surgical procedure or at least delays it.

## Conclusion

Statistically and clinically, greater improvement was observed in the experimental group in terms of improving pain, lumbar ROM, back muscle endurance, functional disability, and physical role domain of QOL compared to the routine physical therapy group. Based on the study results, decompression therapy combined with routine physical therapy is superior to routine physical therapy alone for the management of lumbar radiculopathy in the short-term.

## Data Availability

The datasets generated and analyzed during the present study are not publicly available due to limitations of ethical approval involving the patient data and anonymity but are available from the corresponding author on reasonable request.

## Abbreviations

BME: Back muscle endurance
AROM: Active Range of motion
LBP: low back pain
CONSORT: Consolidated standards for reporting trials
ODI-U: Urdu version of the Oswestry disability index
IRB: Institutional review board
BMI: Body mass index
RPT: Routine Physical Therapy
VAS: Visual analogue scale
TENS: Transcutaneous electrical nerve stimulation
NSD: Non-Spinal decompression
MCID: Minimal clinically important difference
MDC: Minimum detectable change
ANCOVA: Analysis of covariance
SF-36: Short Form 36-Item Health Survey
